# Violation of an Evolutionarily Conserved Immunoglobulin Diversity Gene Sequence Preference Promotes Production of dsDNA-Specific IgG Antibodies

**DOI:** 10.1371/journal.pone.0118171

**Published:** 2015-02-23

**Authors:** Aaron Silva-Sanchez, Cun Ren Liu, Andre M. Vale, Mohamed Khass, Pratibha Kapoor, Ada Elgavish, Ivaylo I. Ivanov, Gregory C. Ippolito, Robert L. Schelonka, Trenton R. Schoeb, Peter D. Burrows, Harry W. Schroeder

**Affiliations:** 1 Department of Medicine, Division of Clinical Immunology and Rheumatology, University of Alabama at Birmingham, Birmingham, Alabama, United States of America; 2 Program in Immunobiology, Carlos Chagas Filho Institute of Biophysics, Federal University of Rio de Janeiro, Rio de Janeiro, Brazil; 3 Department of Microbiology, University of Alabama at Birmingham, Birmingham, Alabama, United States of America; 4 Department of Pediatrics, University of Alabama at Birmingham, Birmingham, Alabama, United States of America; 5 Department of Genetics, University of Alabama at Birmingham, Birmingham, Alabama, United States of America; 6 Genetic Engineering Division, National Research Center of Egypt, Ad Doqi, Egypt; INRA, FRANCE

## Abstract

Variability in the developing antibody repertoire is focused on the third complementarity determining region of the H chain (CDR-H3), which lies at the center of the antigen binding site where it often plays a decisive role in antigen binding. The power of VDJ recombination and N nucleotide addition has led to the common conception that the sequence of CDR-H3 is unrestricted in its variability and random in its composition. Under this view, the immune response is solely controlled by somatic positive and negative clonal selection mechanisms that act on individual B cells to promote production of protective antibodies and prevent the production of self-reactive antibodies. This concept of a repertoire of random antigen binding sites is inconsistent with the observation that diversity (D_H_) gene segment sequence content by reading frame (RF) is evolutionarily conserved, creating biases in the prevalence and distribution of individual amino acids in CDR-H3. For example, arginine, which is often found in the CDR-H3 of dsDNA binding autoantibodies, is under-represented in the commonly used D_H_ RFs rearranged by deletion, but is a frequent component of rarely used inverted RF1 (iRF1), which is rearranged by inversion. To determine the effect of altering this germline bias in D_H_ gene segment sequence on autoantibody production, we generated mice that by genetic manipulation are forced to utilize an iRF1 sequence encoding two arginines. Over a one year period we collected serial serum samples from these unimmunized, specific pathogen-free mice and found that more than one-fifth of them contained elevated levels of dsDNA-binding IgG, but not IgM; whereas mice with a wild type D_H_ sequence did not. Thus, germline bias against the use of arginine enriched D_H_ sequence helps to reduce the likelihood of producing self-reactive antibodies.

## Introduction

Lymphocyte antigen receptors can be positioned on an innate versus adaptive axis that reflects the presumed contributions of natural versus somatic selection. the innate receptor repertoire is viewed as limited, restricted by germline sequence, and thus dependent on natural selection for its recognition of more highly conserved ligands [1]. In contrast, the large and presumed unrestricted immunoglobulin repertoire is viewed as being randomly generated by VDJ recombination and N addition, and somatically selected, permitting recognition of a limitless array of antigens [2–4]. These views have created a theoretical framework for the study of self/non-self discrimination where the innate system responds to the physical or chemical properties of conserved ligands [3]; whereas the adaptive system is focused on discriminating between diverse antigens on a case-by-case basis [3,5]. This concept has major translational implications because it leads investigators seeking to understand or control patterns of responses against individual exogenous or self antigens to focus on the somatic mechanisms of repertoire selection that act on individual cells.

Challenges to this widely-held view derive from increasingly robust compilations of sequences that permit assessment of the actual range of diversity of antibody repertoires [6–10]. These studies have revealed specific biases in the amino acid composition of antigen binding site repertoires. These biases are most striking for CDR-H3, which is the direct product of VDJ rearrangement and N addition and thus constitutes the most diverse portion of the initial antibody repertoire [6,11]. Because CDR-H3 lies at the center of the antigen binding site, its sequence and structure often has a major effect on the binding characteristics of the individual antibody.

Tyrosine, tryptophan and arginine are the amino acids that typically make the greatest contribution to binding affinity at protein ligand-receptor interfaces [12]. In all jawed vertebrates, both tyrosine and, to a lesser extent, tryptophan are found more frequently in CDR-H3 loops than chance alone would dictate; however, arginine is not [6,11]. This raised the possibility that natural selection (Darwinian) or somatic selection (clonal or Burnetian [13]) might be operating categorically on the immunoglobulin repertoire to limit the use of specific amino acids.

**Fig 1:**
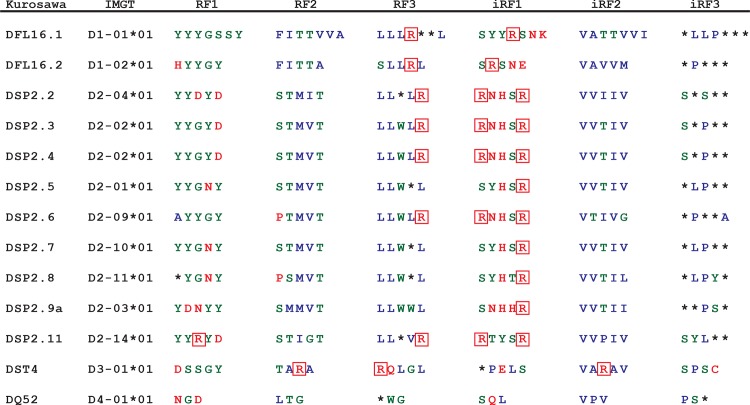
Amino acid content encoded in the six reading frames of the 13 wild type BALB/c D_H_ gene segments. In the RFs generated by DNA deletion, RF1 is enriched in tyrosine and glycine (neutral amino acids, green), RF2 encodes hydrophobic amino acids (blue), and RF3 encodes mostly hydrophobic amino acids and stop codons (*). Amino acids encoded in the RFs generated by DNA inversion (iRFs) are similar to their deletion counterparts except for iRF1 in which charged amino acids (red), specially arginine (framed Rs), are predominant.

To assess *in vivo* whether the preference for tyrosine in CDR-H3 reflects antigen-driven somatic selection, we previously analyzed the repertoires of developing B lineage cells in BALB/c bone marrow [11]. While the prevalence of tyrosine rose during the transition from progenitor (Hardy fraction B) to mature, recirculating B cells (Hardy fraction F), supporting a role for somatic selection; enrichment for tyrosine over arginine was established at the earliest stages of B cell development studied, well before the cell surface expression of IgM, suggesting that the preference for tyrosine was already present at the time of VDJ recombination. These observations led us to re-evaluate the coding sequences of D_H_ gene segments in jawed vertebrates [6,11]. The composition and pattern of rearrangement the 13 D_H_ in BALB/c mice is illustrative (**Fig. 1**). Each BALB/c D_H_ has access to six potential open reading frame sequences: three by deletion and three by inversion [14,15]. Of the 78 possible sets of D_H_ amino acid sequences, only 14 (18%) include one or more tyrosine, whereas 22 (28%) include one to two arginines (**Fig. 1**). However, multiple mechanisms act to favor the use of tyrosine-enriched RF1 over arginine-enriched RFs [14–16] in nascent Ig H chain genes. For example, half of the D_H_ encoded arginines are in iRF1s, but these are used only rarely (<0.01% of rearrangements) [17]. These reading frame amino acid usage biases in D_H_ and CDR-H3 content are conserved from shark to human [6]. This suggested to us that natural selection was operating on immunoglobulin diversity gene segments to restrict and control their evolution in such a way as to influence the composition and range of diversity of immunoglobulin antigen binding sites.

**Fig 2:**
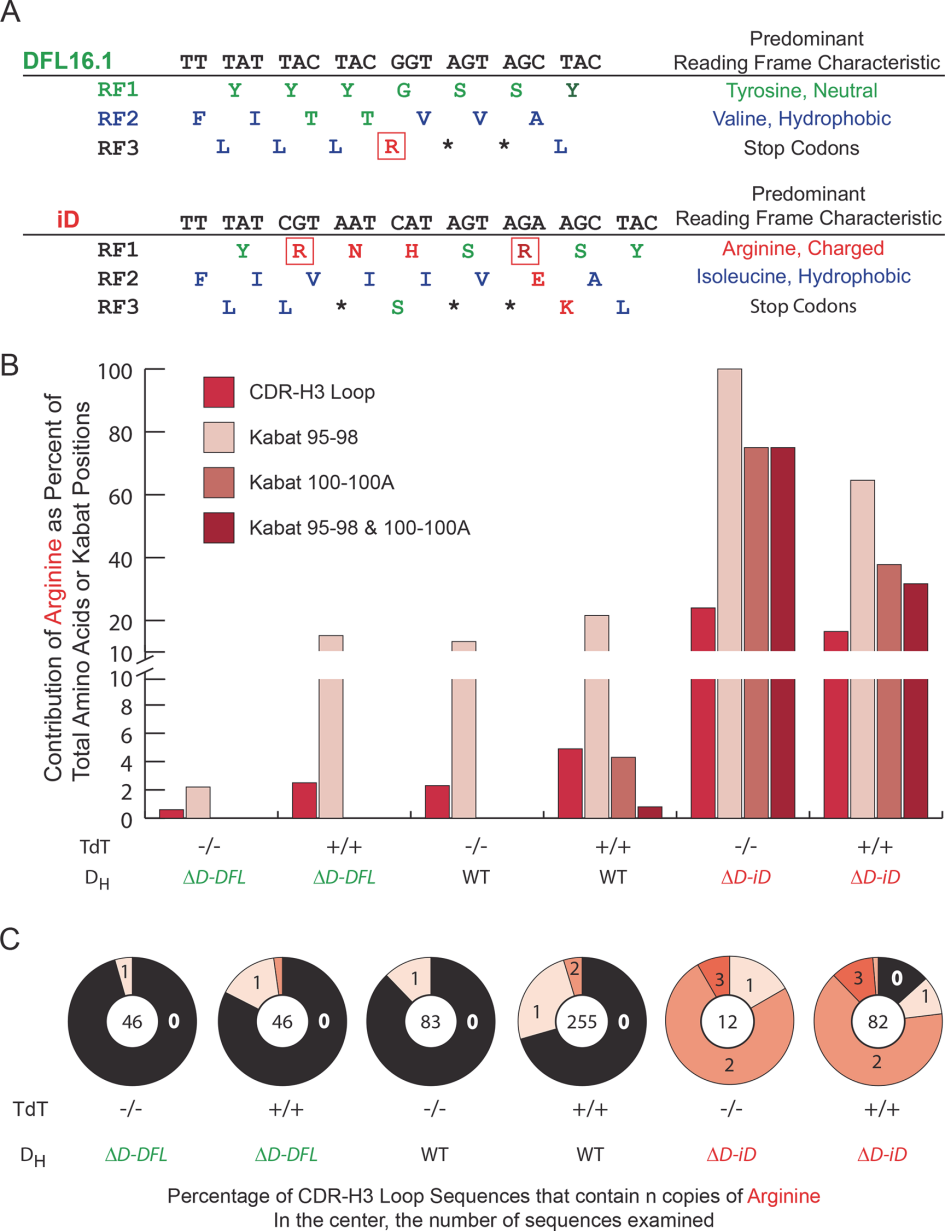
Distribution of arginine in CDR-H3 as a function of D_H_ sequence. **A**) Germline sequences of D_H_ genes and translation in reading frames (RF) 1–3. DFL16.1 RF1 encodes mostly neutral amino acids (green) while iD RF1 encodes mostly charged amino acids (red). In both genes, RF2 encodes hydrophobic amino acids (blue) and RF3 contains stop codons (*). **B**) Percentage CDR-H3 loop sequences containing arginines in Kabat position 95–98, 100–100a, or both. **C**) Distribution of arginines per CDR-H3 sequence in 8 week bone marrow immature B cells as a function of D_H_ content and the presence or absence of TdT. The sequences from TdT-sufficient ΔD-DFL, WT and ΔD-iD mice, and TdT deficient WT mice were previously reported, but reanalyzed for this work [18,34]. The inner circle contains the total number of sequences examined.

To test the relative contributions of natural versus somatic selection to CDR-H3 sequence content, we previously targeted the D_H_ locus of BALB/c ES cells to generate a panel of BALB/c mouse strains whose D_H_ loci had been modified to contain either a single normal or single altered D_H_ [18]. The ΔD-DFL IgH allele uses only wild type (WT) *DFL16*.*1*, which does not encode an arginine in RF1 (Fig. 2A). The ΔD-iD allele inverts *DSP2*.*2*, shifting the two arginines from iRF1 into RF1. Accordingly, in pre-B cells through mature B cells, the repertoire produced by the ΔD-iD allele was enriched for arginine and depleted of tyrosine. Homozygous use of the ΔD-DFL allele yielded normal numbers of peripheral B cells; however, homozygous use of the ΔD-iD allele led to reductions in follicular and recirculating mature B cell numbers with an increase in marginal zone B cell numbers [18]. These subset-dependent changes in B cell numbers mirrored differences in the prevalence of highly charged CDR-H3s in the repertoire of WT mice, suggesting that while somatic selection could not overcome the bias in amino acid usage imposed by Darwinian selection of the sequence of the D_H_, normal processes of Burnetian clonal selection were acting categorically to sort B cells into specific niches based, in part, on the physico-chemical properties of the amino acids at the center of the antigen binding site [18,19].

Molecular evolution of D_H_ content could reflect selection for the ability to respond protectively to foreign antigens and pathogens or selection against the relative facility of generating potentially pathogenic, self-reactive antibodies. In support of the first hypothesis, we previously found that young ΔD-iD mice had reduced antigen-specific antibody responses post vaccination, and increased susceptibility to *Streptococcus pneumoniae* and influenza virus post live challenge; whereas ΔD-DFL mice did not [18,20]. Thus, violation of germline D_H_-imposed CDR-H3 preferences had created an immune deficient phenotype.

However, these positive and negative selection hypotheses are not mutually exclusive. To test the second hypothesis, we set out to determine whether violation of D_H_ sequence preferences could facilitate production of antibodies with potentially pathogenic self-reactivities. Under normal circumstances, BALB/c mice are considered to be resistant to the production of dsDNA-binding antibodies [21,22]. However, arginines in the antigen binding sites of dsDNA binding IgG are often critical for this specificity [23–25]. Thus, we examined mice bearing one or two copies of the arginine-encoding ΔD-iD allele for the production of DNA-binding antibodies. We found that over one year’s time one-fifth of the mice enriched for CDR-H3s that included two or more arginines expressed dsDNA-binding IgG in their blood. This suggests that both hypotheses are correct, and that the molecular evolution of D_H_ sequence may be shaped, at least in part, by the need to provide anticipatory protection against production of potentially pathogenic, self-reactive IgG.

## Materials and Methods

### Ethics statement

All experiments were approved by and performed in compliance with UAB IACUC regulations. Carbon dioxide (CO2) inhalation followed by cervical dislocation was used for euthanasia.

### Mice and blood samples

TdT-deficient BALB/c mice [26] were bred to homozygosity with ΔD-iD or ΔD-DFL BALB/c mice [18] in the UAB vivarium. Cohorts of 10–15 were prepared for each of the following genotypes: ΔD-DFL homozygous, ΔD-DFL/WT heterozygous, WT BALB/c, ΔD-iD homozygous, and ΔD-iD/WT heterozygous. Eight to ten weeks-old mice were bled from the tail vein at monthly intervals. Serum was obtained by centrifugation and stored at -80°C until analysis.

### CDR-H3 sequencing

Immature bone marrow B cells were identified and sorted as described elsewhere [11]. Total RNA isolation, VH7183 specific VDJCμ RT-PCR amplification, cDNA cloning into plasmid vectors, CDR-H3 sequencing, and sequence analysis was performed as previously described [11].

### Anti-DNA ELISA

ELISAs were performed as reported previously [27]. Briefly, 96-well plates (Costar 9018, Corning Incorporated) were treated with poly-L-lysine solution for 2 hours and then coated with DNA sodium salt from calf thymus (D3664-2MG, Sigma-Aldrich). Sera samples (three 1:2 serial dilutions), and HRP-labeled secondary antibodies were diluted in a 1.5% BSA-PBS. Secondary antibodies against mouse IgM (1020-05), IgG (1031-05), IgG1 (1070-05), IgG2a (1080-05), IgG2b (1091-05), and IgG3 (1101-05) were obtained from Southern Biotech (Birmingham, AL, USA). To develop the ELISA we used 100 μL of 1X TMB ELISA substrate solution (eBioscience 00-4201-56, San Diego, CA, USA) per well and incubated 10 minutes in the dark at RT. The reaction was stopped using 50 μL of 2N H_2_SO_4_, read and analyzed in a FLUOstar Omega microplate reader (BMG Labtech, Ortenberg, Germany).

### CLIF assays

*Crithidia luciliae* IFA complete kits (Bio-Rad 30404, Hercules, CA, USA) were used per the manufacturer’s recommendations. Sera samples were diluted 1:10, 1:50, and 1:100 in PBS.

### Kidney histological analysis

Paraffin sections from formalin-fixed kidneys were periodic acid-Schiff-hematoxylin (PAS-H) stained in the Comparative Pathology Laboratory of the University of Alabama at Birmingham Animal Resources Program. Histopathologic examination and morphometric analysis were done by an expert rodent pathologist (TRS) with experimental group identifications concealed. For histomorphometry, images of glomeruli were made with a SPOT Insight digital camera (Diagnostic Instruments) at an objective magnification of 40x. Images were analyzed as previously described [28].

### Limiting dilution analysis

B-1a cells were isolated from the peritoneal cavity and cultivated under limiting dilution conditions in the presence of 30 μg/ml of LPS (*Salmonella typhimurium*, Sigma-Aldrich, St. Louis, MO, USA), using 3 × 10^3^ cells of the S17 stroma cell line as feeder cells per culture as feeder cells [29]. Variable numbers of B-1a cells from each mouse strain in 22 replicates for each cell concentration (10,000; 3,000; 1,000; 300; 100; 18, 6, 2, and 0.66) were cultured to determine the frequency of B-1a clones secreting IgM binding to dsDNA. The growth of dsDNA IgM secreting B cell clones was evaluated by ELISA for the presence of IgM anti-dsDNA in the culture supernatant by ELISA from cells derived from each mouse strain. The percentage of negative cultures was plotted against the B cell number per well, and frequencies of dsDNA specific cells were calculated according to Poisson’s distribution [30].

### FACS analysis

Single cell suspensions of the spleen and bone marrow (BM) from both femurs of each 8 weeks or 12 month old individual mice were prepared, stained and analyzed as described previously [11,31,32].

### BLyS assays

BLyS levels in mouse serum were determined using the BLyS ELISA Kit (Antibodies-online Inc. Atlanta, GA, USA), as instructed by the manufacturer. The optical density (OD) was determined by using a micro-plate reader (set to 450 nm (BMG Labtech). The concentration of BLyS in the serum was calculated using a standard curve, prepared in the same plate and with the appropriate dilution factor.

### Evaluation of B cell proliferation and cell cycle progression

B cell proliferation. Short term intra-peritoneal bromodeoxyuridine (BrdU) injection (1mg/mouse) was used to evaluate rapidly proliferating B cell subsets in the bone marrow. Mice were injected twice at a 12 hours interval, and then sacrificed 24 hours later. BrdU positive cells were detected using antibodies specific for BrdU (FITC BrdU-flow kit; BD Biosciences, San Jose, CA, USA) according to the manufacturer’s protocol. BrdU in the drinking water for seven days was used for long term BrdU incorporation in slowly proliferating mature B cell subsets. The mice received drinking water containing BrdU (1mg/ml; Sigma-Aldrich)/2% sucrose, protected from light and changed each every three days. Single cell suspensions of the bone marrow, spleen, and peritoneal cavity were prepared by filtering through a 70-μm cell strainer (BD Falcon, Franklin Lakes, NJ, USA) and washed twice with 2% FACS buffer. Fixation, permeabilization, and preparation of the cells for flow-cytometric detection of BrdU positive cells were performed according to the FITC BrdU flow kit manufacturer’s instructions (BD Biosciences).

**Cell cycle progression**. BrdU and 7AAD staining was used to evaluate the percentage of B cells in different phases of the cell cycles according to (FITC BrdU-flow kit; BD Bioscience) based on the manufacturer’s protocol (FITC BrdU-flow kit; BD Biosciences). The percentage of each B cell subsets in different cell cycle phases was compared between different genotypes and relative to WT BALB/C.

### Evaluation of the prevalence of apoptotic cells

The percentage of B cells undergoing apoptosis in the various B cell subsets of the bone marrow and spleen was determined based on the dual expression of Propidium Iodide (PI) and Annexin V. All studies were performed in accordance with the manufacturer’s instructions provided by the FITC Annexin V Apoptosis Detection Kit II (BD Biosciences).

### Statistical analysis

Two tailed Student t test, ANOVA, the Wilcoxon/Kruskal-Wallis (rank sums) test, Levene’s test for homogeneity of variance, non-parametric median test, or Pearson Product Moment Correlation were used to analyze the difference between populations. Analysis was performed with JMP version 10 (SAS, Cary, NC, USA) or Sigmaplot version 9 (Systat Software, San Jose, CA, USA).

## Results

### The sequence of the D_H_ largely controls the arginine content of the CDR-H3 loop in TdT-sufficient mice

A preference for arginines in Kabat CDR-H3 positions 95–98 and 100–100A characterizes many dsDNA binding Igs [24,33]. To assess the relative contribution of N addition versus D_H_ sequence to CDR-H3 loop arginine content, we combined for analysis our previously published sequences from immature bone marrow B cells (Hardy Fraction E) of WT, homozygous ΔD-DFL, homozygous ΔD-iD, and TdT-deficient BALB/c mice [18,34] with new sequences from TdT-deficient ΔD-DFL and ΔD-iD mice (**S1 and S2 Tables**).

The sequence of the D_H_ was observed to play a greater role than N addition in controlling both the prevalence and position of arginines in CDR-H3 loops. In WT and ΔD-DFL mice with or without TdT, less than 5 percent of CDR-H3s contained more than one arginine, and less than one percent of the CDR-H3s contained an arginine within both Kabat positions 95–98 and 100–100A. In contrast, more than 80% of sequences from ΔD-iD mice contained two or more arginines in CDR-H3, and 75% of the TdT-deficient ΔD-iD mice and 32% of the TdT-sufficient ΔD-iD mice encoded arginines at both Kabat positions (**Fig. 2**).

### BALB/c mice using the ΔD-iD allele are prone to produce DNA-specific IgG, but not IgM

**Fig 3:**
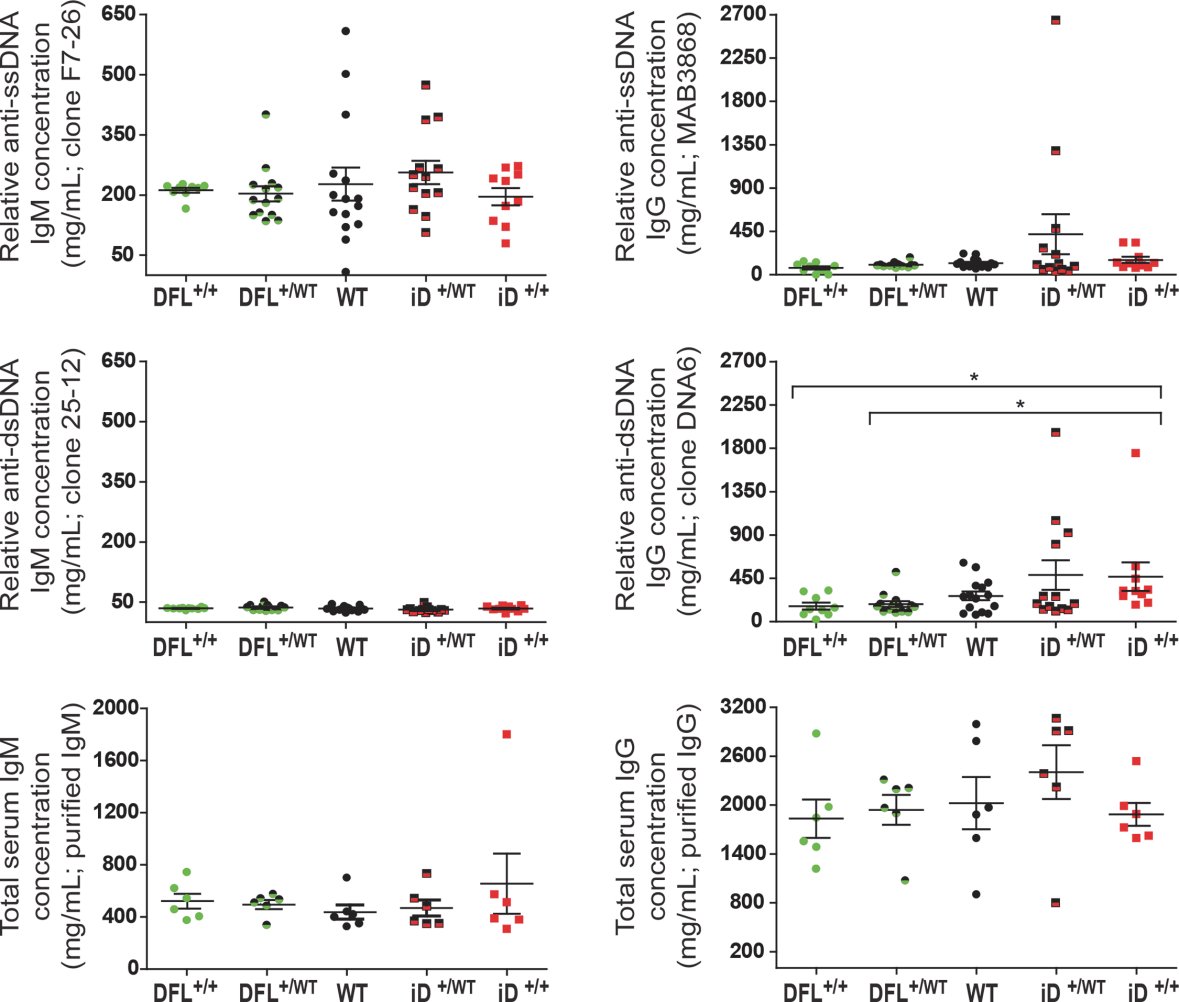
ssDNA- and dsDNA-specific, as well as total IgM and IgG production at 12-months of age. Statistical differences between groups were determined by one-way ANOVA with a Dunn’s multiple comparison post-test. Comparisons between homozygous ΔD-iD and homozygous and heterozygous ΔD-DFL achieved statistical significance (* p < 0.05).

**Fig 4:**
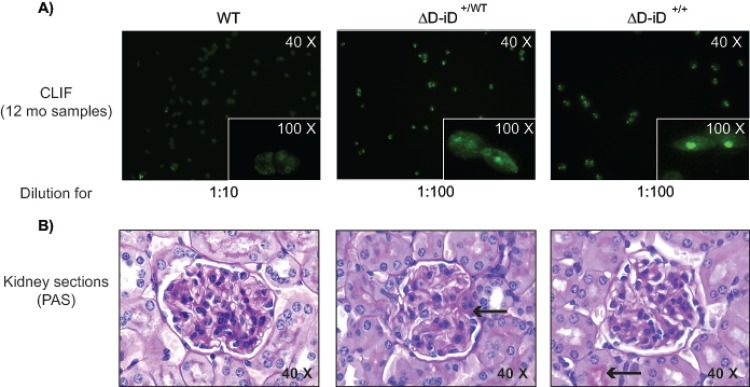
Histologic evaluation of dsDNA activity in the serum and its effect on renal tissue. **A**) Representative CLIF assay images showing positive staining in ΔD-iD, but not WT, samples. Original magnification on kidney and CLIF assay images are at 40X, inserts showing representative staining for the CLIF assay are at 100X. The positivity in CLIF assays confirms the DNA-specific reactivity of ELISA-tested samples. **B**) PAS-H stained kidney sections from 12 month old mice. Minor fibrin deposition was observed in some ΔD-iD kidneys (center and right panels, arrows), but not WT (left panel) or ΔD-DFL (not shown).

To test whether increased use of an arginine-encoding RF would enhance the likelihood of producing DNA binding antibodies in otherwise un-manipulated mice, we created cohorts of 10–15 BALB/c mice carrying WT, ΔD-DFL and ΔD-iD IgH alleles. From 2 months to 12 months of age, we then collected sera at monthly intervals. To avoid possible confounding effects of T cells with receptors lacking N nucleotides, all mice were TdT-sufficient. The panel of 12-month sera was tested by ELISA for antibodies binding to single stranded DNA (ssDNA) and to dsDNA (**Fig. 3**). Samples from 12-month-old homozygous and heterozygous ΔD-iD mice that were positive for ssDNA- and dsDNA-specific IgG by ELISA were also positive for kinetoplast and nuclear staining in CLIF assays (1:100 dilution, **Fig. 4A**). By contrast, WT and ΔD-DFL sera samples were negative even at the lowest dilution (1:10). The possibility that an increase in the total concentration of sera IgM and IgG would create an artifact by increasing the natural antibody representation in ΔD-iD mice was ruled out by the similarity in total IgM and total IgG concentration among all the groups (**Fig. 3**).

At 12 months of age we detected increased serum levels of IgG antibodies binding to dsDNA in 20–40% of mice containing either one or two ΔD-iD alleles, suggesting that the effect of the ΔD-iD allele was phenotypically dominant (**Fig. 3**). Mice lacking the ΔD-iD allele showed only low levels of these antibodies.

### Glomerulonephritis was not observed in ΔD-iD BALB/c mice expressing high levels of dsDNA-binding IgG

To assess whether in these otherwise normal mice with high levels of dsDNA-binding antibodies developed glomerulonephritis, kidneys were collected at 12 months of age and fixed in formalin for histological analysis [periodic acid-Schiff-hematoxylin (PAS-H) staining]. Analysis of histomorphometry data did not indicate significant differences in glomerular area, PAS staining, or nuclear area. Minor fibrin deposition was observed in some ΔD-iD kidneys, but not WT or ΔD-DFL. One ΔD-DFL homozygous mouse with a low concentration of dsDNA-specific antibodies had moderate glomerular capillary thrombosis. Most glomeruli of the remaining mice were normal (**Fig. 4B**), although some mice had a few glomeruli with slight to mild mesangial expansion. This was observed in mice of all genotypes and in mice with high as well as low antibody titers. Mild mesangial expansion was considered to be within normal limits for mice of this age.

### No increase in the number of B-1a B cells producing dsDNA-binding antibodies

**Fig 5:**
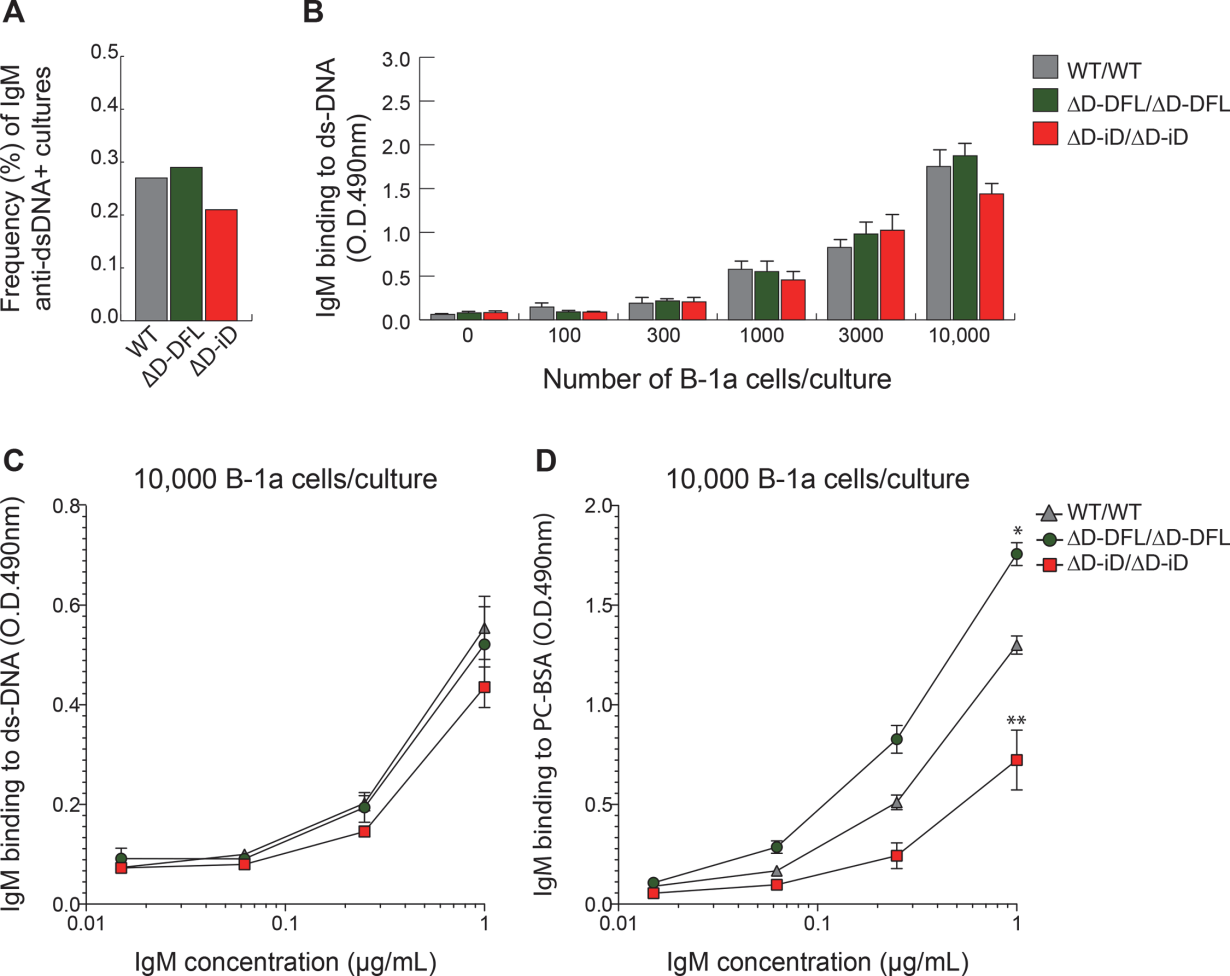
Limiting dilution analysis of dsDNA binding production by PerC B-1a B cells. Frequency of peritoneal cavity B-1a cells expressing dsDNA-binding BCR by genotype. PerC B-1a cells were sorted and cultured under polyclonal stimulation for IgM secretion. The frequency of B-1a cells secreting IgM anti-dsDNA *in vitro* was estimated by LDA. **A)** Histogram bars show the frequency, calculated by the Poisson distribution, of culture supernatants containing IgM binding to dsDNA. **B)** O.D. values of individual culture with variable numbers of B-1a cells. **C)** Evaluation of anti-dsDNA binding of culture supernatants containing 10,000 B-1a cells and similar amount of total IgM per culture. **D)** Binding to non-related antigen (phosphorylcholine—PC) using the same culture supernatants to control for the specificity of the assay. Significantly different from WT: *, P ≤ 0.05; **, P ≤ 0.01.

Most of the IgM present in normal mouse serum consists of natural antibodies, which are commonly produced by the B-1a B cell subset. Since the levels of IgM anti-dsDNA antibodies are established as early as 2 months of age and remain constant until 12 months of age regardless of the mouse strain, we tested whether the B-1a cells bearing BCR with DNA binding characteristics would follow the same pattern observed in the serum. We isolated B-1a cells from the peritoneal cavity of homozygous two month old WT, ΔD-DFL and ΔD-iD mice, performed a limiting dilution analysis [29] and tested for DNA binding. No differences were observed in the prevalence of B1-a cells producing dsDNA-binding IgM (**Fig. 5**).

To gain insight into the quality of dsDNA binding antibodies secreted by B-1a cells *in vitro* following polyclonal stimulation, we evaluated the binding capacity of dsDNA-specific IgM in culture supernatants containing 10,000 B-1a cells from each mouse strain. All groups showed the same level of reactivity to dsDNA. To control the specificity of the assay we also tested the same supernatants against a non-related antigen (phosphorylcholine—PC). All the supernatants were tested at the same total IgM concentration. In accordance with our previous study [35], we found that binding to PC was significantly decreased in B-1a culture supernatants derived from ΔD-iD mice when compared to the WT and ΔD-DFL controls. These observations suggested that the IgM antibodies generated by the different mouse strains appeared to display the same relative affinity to dsDNA.

### Once initiated, dsDNA IgG binding titers increased with age

**Fig 6:**
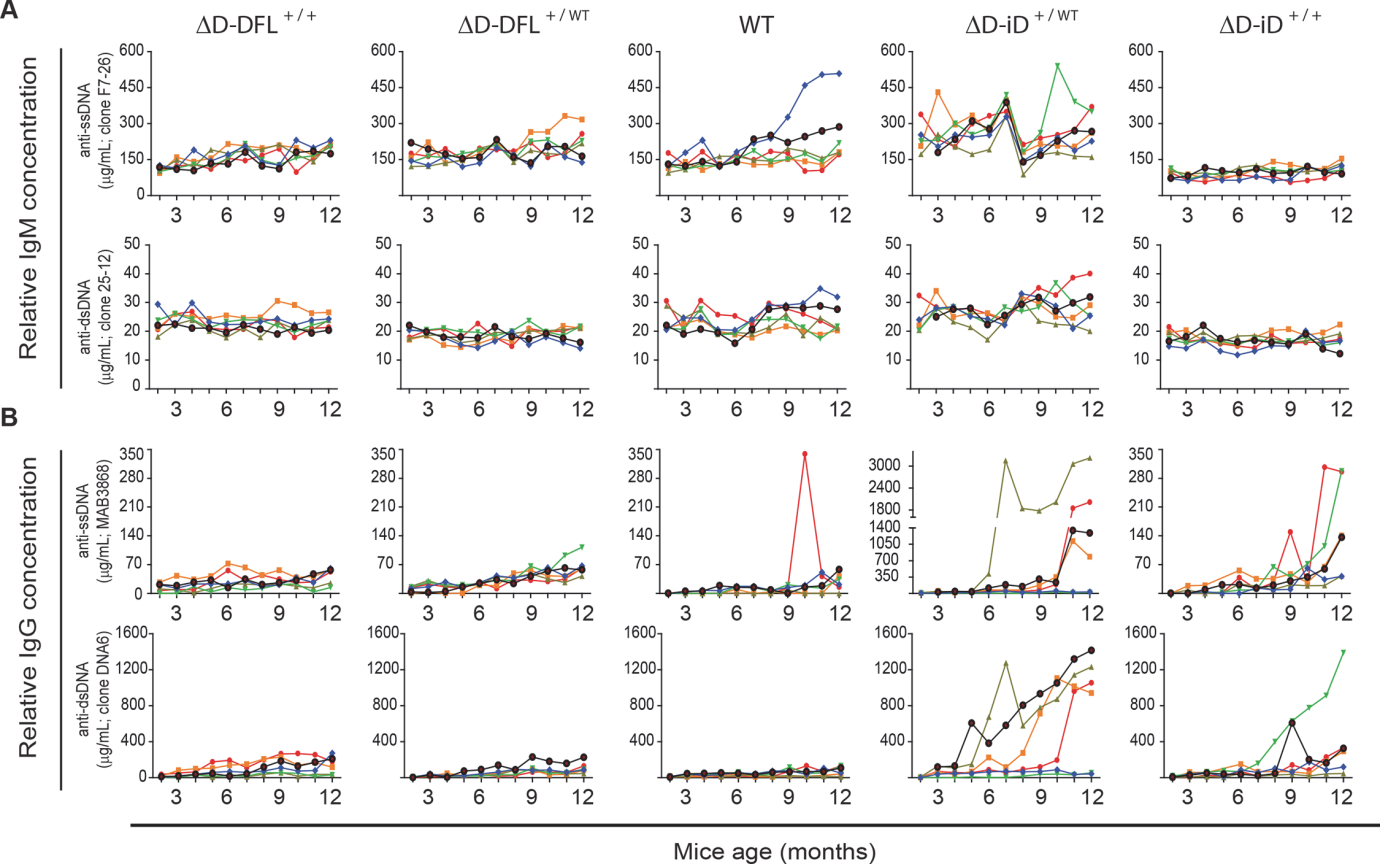
dsDNA binding antibody production as a function of D_H_ genotype and time. WT and ΔD-DFL mice had low and stable ssDNA- and dsDNA-specific **A)** IgM and **B)** IgG levels (columns 1–3). In both homozygous and heterozygous ΔD-iD mice, 20–40% of the mice produced ssDNA- and dsDNA-specific IgG, and it continued to increase in concentration after onset (columns 4–5). Only ΔD-iD heterozygote mice showed transient peaks of dsDNA-specific IgM production. Each plotted line represents the 10-month, DNA-specific antibody production kinetics for individual mice. Concentration of DNA-specific antibodies in sera was calculated using a standard curve of ssDNA- or dsDNA-specific monoclonal antibodies.

**Fig 7:**
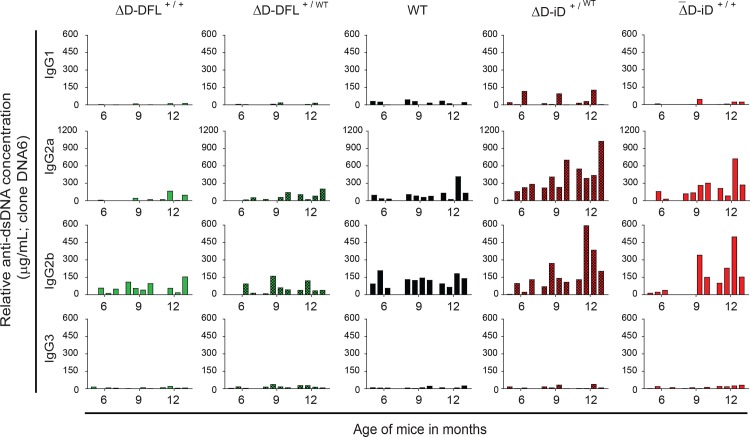
dsDNA-specific IgG isotype levels in 6, 9, and 12 months sera from the four highest responders at 12 months. In the mice containing the ΔD-iD allele, the dsDNA-specific IgG response is composed primarily of IgG2a and IgG2b isotypes.

We selected samples for serial analysis from each genotypic group with the four highest and the two lowest levels of dsDNA-specific IgG. For IgM, we found that the production of ssDNA- and dsDNA-specific antibodies was low throughout the 10-month period of observation (**Fig. 6A**). Production of IgG antibodies binding to ssDNA and to dsDNA (**Fig. 6B**) in WT mice and mice containing the ΔD-DFL allele showed steady and similarly low levels during the 10 month analysis (**Fig. 6B**, columns 1–3). In contrast, increased serum ssDNA- and dsDNA-specific IgG levels were detected in ΔD-iD homozygous and heterozygous mice starting at 8–9 months and 4–5 months respectively (**Fig. 6B**, columns 4–5), and were sustained for 3 or more months. In homozygous and heterozygous ΔD-iD mice positive for dsDNA-specific IgG, serum levels of IgG to dsDNA were 10-14-fold higher than the average for wild type BALB/c, while ssDNA-specific IgG levels were 3-32-fold higher. The antibodies to dsDNA in samples from 6, 9 and 12 months were primarily of the IgG2a and IgG2b isotypes (**Fig. 7**) suggesting a process that requires T cell help and the formation of germinal centers for Ig class switching to occur.

### No increase in serum BLyS levels in mice using the ΔD-iD allele

**Fig 8:**
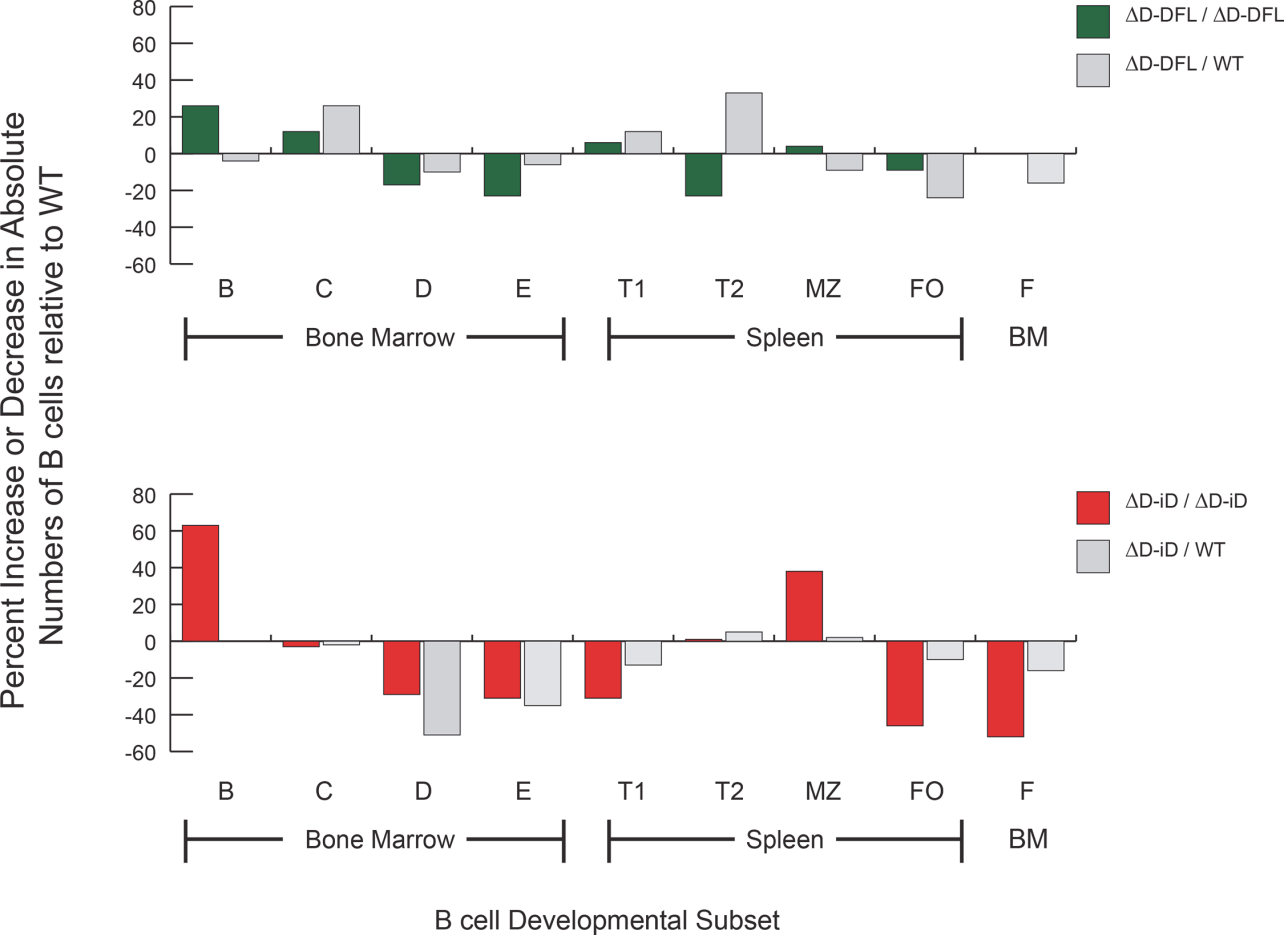
Divergence in the absolute numbers of B lineage subpopulations from the bone marrow, spleen, and peritoneal cavity of homo- and heterozygous ΔD-DFL and ΔD-iD mice relative to their littermate controls. Percent loss or gain in homo- and heterozygous ΔD-DFL (**top**) and ΔD-iD (**bottom**) mice relative to wild type littermate controls in the average absolute number of cells in bone marrow fractions B (CD19^+^ CD43^+^ HSA^+^ BP-1^-^), C (CD19^+^ CD43^+^ HSA^+^ BP-1^+^), D (CD19^+^ CD43^-^ IgM^-^ IgD^-^), E (CD19^+^ CD43^-^ IgM^+^ IgD^-^), and F (CD19^+^ CD43^-^ IgM^lo^ IgD^hi^); and in splenic transitional T1 (CD19^+^ AA4.1^+^ sIgM^hi^ CD23^-^), T2 (CD19^+^ AA4.1^+^ sIgM^hi^ CD23^+^), marginal zone (MZ, CD19^+^ CD21^hi^ CD23^lo^), and mature (M, CD19^+^ CD21^lo^ CD23^hi^) B-cell subsets. The distribution of B cell numbers by subset in homozygous ΔD-DFL and ΔD-iD mice has been previously published [31,32] and is included for clarity. The standard error of the mean of each B lineage subpopulation for the littermate controls averaged approximately 11% of the absolute number of cells in each subpopulation. For ΔD-DFL and ΔD-iD, the standard error of the mean is shown as an error bar. ‘*’, p ≤ 0.05 and ‘****’, p < 0.0001.

Homozygous ΔD-iD and ΔD-DFL mice both exhibit B cell lymphopenia in Hardy fractions D and E in the bone marrow [31,32]. Homozygous ΔD-iD mice also exhibit B cell lymphopenia in the follicular B cell subset in the spleen and recirculating, mature Hardy Fraction F in the bone marrow (**Fig. 8**) [31,32]. To assess the effect of heterozygosity on B cell numbers, we re-examined previously published [36] and unpublished studies of heterozygous D-altered BALB/c mice (**Fig. 8**). By focusing on the percent increase, or decrease, in absolute B cell numbers when compared to wild type, this analysis emphasizes relative differences in B cell numbers at specific B cell developmental stages. With the exception of the follicular B cell subset that exhibited a 25% reduction in B cell numbers (p<0.05), the absolute numbers of B cells in bone marrow and splenic subsets in the heterozygous ΔD-DFL/WT mice were statistically indistinguishable from those in WT/WT littermate controls. Unlike their ΔD-DFL counterparts, heterozygous ΔD-iD/WT mice exhibited reductions in the absolute number of Hardy Fraction D and E. However, among the mature splenic and bone marrow B cell fractions, heterozygous ΔD-iD/WT mice also exhibited normalization of B cell numbers.

**Fig 9:**
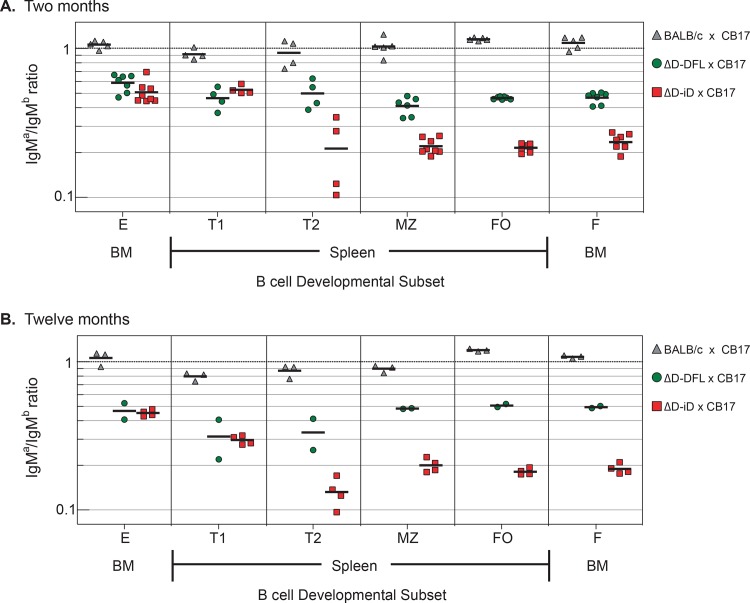
B cells bearing a D_H_-altered allele are disfavored compared to a wild-type repertoire. Flow cytometric analysis indicates the relative proportion of B cells expressing a germline CB17 IgH^b^ allele versus a germline BALB/c IgH^a^ or D_H_-altered allele (ΔD-DFL or ΔD-iD, IgH^a^). Results are shown for a heterozygous F1 wild-type (CB17 x BALB/c) mouse and a heterozygous (CB17 x ΔD-DFL or CB17 x ΔD-iD) D_H_-altered mouse. **A)** Mice were 2 months and **B)** 12 months old. The cells examined are bone marrow derived immature Hardy Fr. E and mature Fr. F, and spleen derived transitional T1, T2; Marginal Zone (MZ) and Follicular (FO) B cells.

To further assess the effect of D_H_ allele heterozygosity on B cell numbers; WT, ΔD-DFL and ΔD-iD mice were crossed with CB17 mice, which are BALB/c congenic for the IgH^b^ H chain allele. Using monoclonal antibodies that can distinguish between the surface expression of BALB/c IgH^a^ versus C57BL/6 IgH^b^, the ratio of IgH^a^ to IgH^b^ staining was determined in bone marrow Hardy Fractions E and F, and in splenic T1, T2, MZ and FO B cells. Mice with WT D_H_ exhibited a 1:1 ratio of mature and follicular B cells at two and 12 months of age (**Fig. 9**). The ΔD-DFL and ΔD-iD alleles disadvantaged their host B cells equally among newly arisen B cells (Hardy Fraction E and splenic T1). Further discrimination against use of the ΔD-iD allele was observed in the transition from T1 to T2. The ratio of use of these alleles then remained unchanged in the marginal zone and the follicles. This pattern was maintained at twelve months of age.

BLyS (B Lymphocyte Stimulator) plays a critical role determining the available homeostatic ‘space’ for mature naïve B cells [37]. dsDNA binding antibody production flares that occur following B cell depletion therapy in human lupus patients correlate with elevations of BLyS and are attributed to BLyS influenced increased survival of B cells expressing self-reactive BCRs [37,38]. For each of the six mice of each of the five D_H_ genotypes where DNA-binding was evaluated serially (**Fig. 6**), we measured serum BLyS levels at three and ten months of age. BLyS levels were similar regardless of D_H_ genotype (data not shown). A Pearson Product Moment Correlation analysis testing the relationship between BLyS and anti-dsDNA levels at 10 months of age was performed on the samples from six mice from each of the five D_H_ genotypes that were serially followed for the presence of dsDNA binding IgM and IgG in the sera. No correlation was found.

### Lymphopenia in the D-altered mice reflects delays in cell cycle progression, not apoptosis

**Fig 10:**
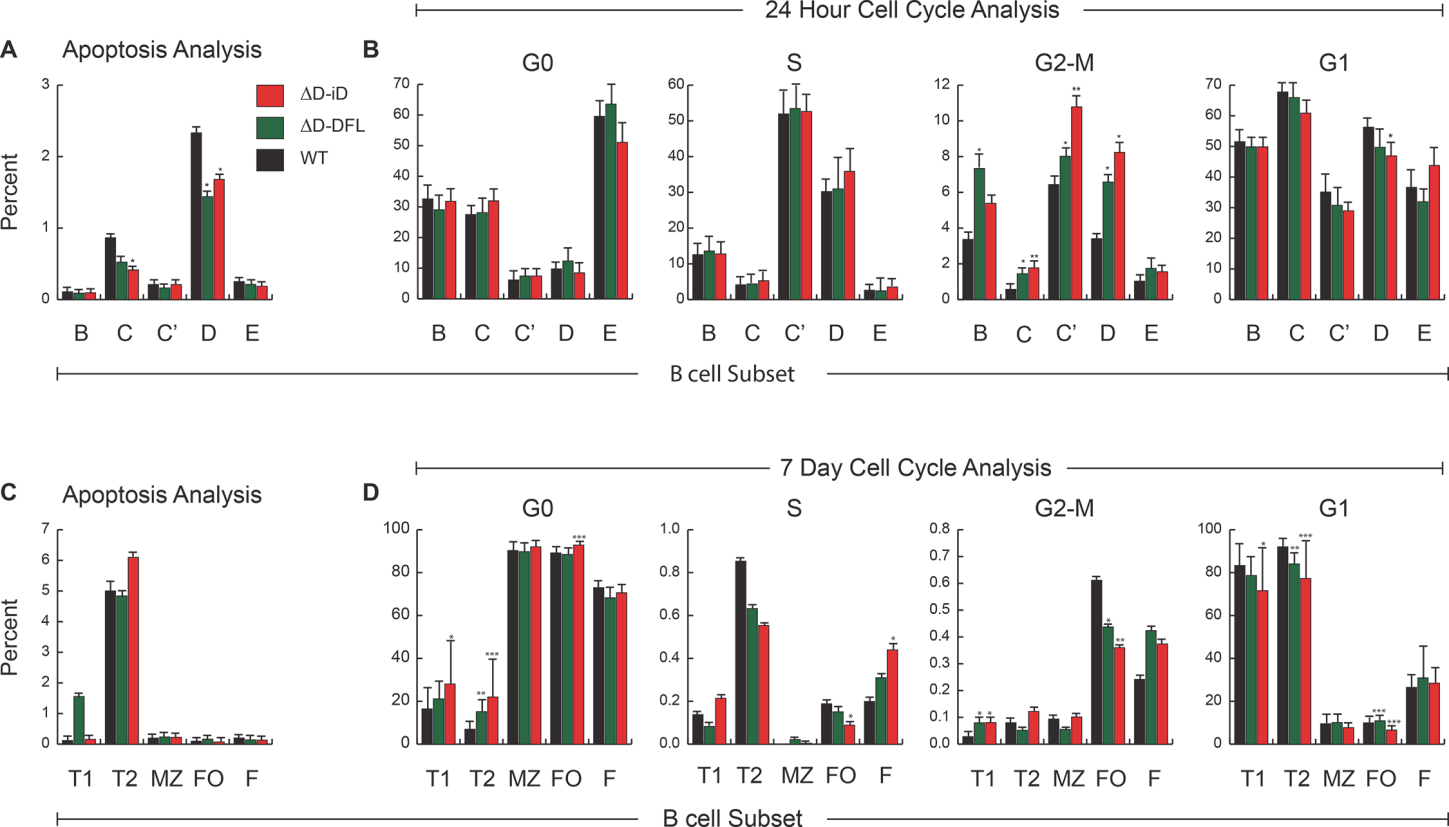
Analysis of the prevalence of cells undergoing apoptosis or cell division reveals that D_H_ altered B cells are less likely to undergo programmed cell death, but more likely to be quiescent. **(A)** *Percentage of developing Hardy Fractions B to E B cells in late stage apoptosis in the bone marrow*. A total of 14 WT BALB/c, 11 ΔD-DFL and 14 ΔD-iD mice were examined. Late apoptotic cells co-expressed PI and Annexin V. **(B)** *Percentage of developing Hardy Fractions B to E B cells in G0 (quiescent cells)*, *S (actively synthesizing DNA)*, *G2-M (mitosis) and G1 (post-mitotic cells) as assessed by differential straining for BrdU and 7AAD*. A total of 6 WT, 11 ΔD-DFL and 10 ΔD-iD BALB/c mice were studied. **(C)** *Percentage of maturing or mature B cell subsets in late stage apoptosis in the spleen or bone marrow*. T1, T2, MZ and FO cells were studied in the spleen, Hardy Fraction F was studied in the bone marrow. A total of 10 WT, 11 ΔD-DFL and 14 ΔD-iD BALB/c mice were studied. **(D)** *Percentage of maturing or mature B cell subsets in G0*, *S*, *G2-M and G1 as assessed by differential straining for BrdU and 7AAD*. A total of 6 WT, 8 ΔD-DFL and 5ΔD-iD BALB/c mice were studied. The Standard error of the mean is presented as bars above each column. Significance values are listed as ‘*’, p ≤ 0.05; ‘**’, p ≤ 0.01; ‘***’, p < 0.001; and ‘****’, p < 0.0001.

The decline in the numbers of B cells bearing altered D_H_ alleles suggested that cells that used CDR-H3s enriched for ‘disfavored’ amino acids were either being eliminated or were not being properly expanded. In two months old mice bearing either WT or altered D_H_, we assessed the percent of cells within each bone marrow and splenic subset that stained with both propidium iodide (PI) and Annexin V, markers of late apoptosis (**Fig. 10A,C**). Contrary to expectations, among the bone marrow pre B cells in Hardy Fractions C and D a smaller proportion of cells from the D_H_ altered mice expressed these markers of apoptosis when compared to WT controls. No significant difference in the prevalence of apoptosis was found in T1, T2, MZ or FO B cells.

To address the issue of expansion, we then performed BrdU kinetic cell cycle analyses on B cells in the bone marrow (BrdU administration by injection) and spleen (BrdU administration by means of drinking water). When compared to WT, there was a significant increase in the percent of cells at the post-mitotic stage (G2-M) in Fractions C, C’ and D of ΔD-iD bone marrow (**Fig. 10B**). In support of the view that cell cycle progression had been retarded, we observed a compensatory decrease in the percent of cells in fraction D that had undergone at least one round of division in a 24-hour period (G1). This pattern was also apparent, although to a lesser extent, in the ΔD-DFL mice.

The percentage of cells undergoing cell division in the more mature B cell subsets in the spleen and bone marrow is much smaller (**Fig. 10D**). However, we again observed a relative block in cell cycle progression. In particular, the transitional B cell subsets that included T2 exhibited a trend for decreased entry into mitosis with a statistically significant decrease in the percentage of cells that had completed at least one round of cell cycle division in a week’s time (G1). In the follicles, the percentage of ΔD-iD bearing cells that had not divided (G0) was also increased. Thus, the greater the divergence of the BCR repertoire from normal, the more quiescent the B cell subpopulation.

## Discussion

### The composition of CDR-H3 is biased across evolution

Although the global composition of the antibody repertoire differs across individuals and between species, we and others have observed biases in the sequence, structure and hydrophobicity of V domain components that are conserved across evolution [6,39–43]. In order to assess the selective mechanisms and the biologic impact of this form of stereotypy in CDR-H3, we previously generated a panel of D-altered mice [18] containing IgH loci limited to one wild type or one altered D_H_. B cells in D-altered mice undergo the normal processes of VDJ recombination and N addition to create large, diverse, and polyclonal H chain repertoires. The representation of V gene segment utilization appears minimally affected by these changes in CDR-H3 content [18]. D-altered repertoires contain an alternative CDR-H3 gradient that can include an amplified representation of categories of sequences that are present normally but can be difficult to study due to their low prevalence. B cells using D-altered alleles pass through all the normal checkpoints of B cell development. They respond to antigenic challenge, and can undergo class switching, somatic hypermutation, and affinity maturation. The only difference between the mice is the D_H_ component that generates a bias in amino acid usage in CDR-H3, which lies at the center of the antigen binding site.

### Half of the arginines in CDR-H3 derive from N addition and half from germline D_H_ sequence

Study of our panel of D-altered mice allowed us to determine that approximately half of the arginines in the CDR-H3 region of the BCR of immature B cells in BALB/c mice derive from N addition and half from germline D_H_ sequence (**Figs. 1 and 2**). The prevalence of arginine in TdT-sufficient WT CDR-H3 loops (4.9%) was twice that of TdT-sufficient ΔD-DFL (2.5%, p = 0.04) or TdT-deficient WT (2.3%, p = 0.06) mice (**Fig. 2**). Less than one percent of CDR-H3s from TdT-deficient ΔD-DFL mice encoded arginine (p = 0.006 vs WT). These arginines derived from use of RF3 or palindromic (P) junctions. Conversely, arginine comprised approximately one-sixth of the amino acids in CDR-H3s from TdT-sufficient ΔD-iD mice, and one-fourth (p<0.0001 vs WT) of the amino acids in CDR-H3s from TdT-deficient ΔD-iD mice (**Fig. 2**). The higher prevalence of arginine in TdT-deficient ΔD-iD mice reflected a decrease in exonucleolytic nibbling in the absence of TdT due to enhanced rearrangement at sites of VDJ microhomology [34,44]. These findings allow us to confirm that the prevalence of arginine in CDR-H3 reflects the non-random distribution of this amino acid by D_H_ reading frame (**Figs. 1 and 2**). By violating the effects of natural selection on D_H_ reading frame amino acid preferences we created mice whose developing B cells express greatly increased numbers of immunoglobulins with CDR-H3s containing two or more arginines in positions that have been shown to facilitate binding to dsDNA (**Fig. 2**) [24,33].

### The effect of increasing D_H_ arginine content on dsDNA binding autoantibody production appears aged-influenced and a Mendelian dominant, albeit with incomplete penetrance

In spite of enhanced usage of arginine, in both homozygous and heterozygous ΔD-iD mice the production of B cells that express or secrete dsDNA-binding IgM antibodies above the wild-type baseline appears to have been normally suppressed (**Fig. 3**). These findings suggest that the clonal selection mechanisms of anergy, receptor editing, and cell death that normally prevent the production and survival of B cells bearing potentially pathogenic self-reactive BCR in the bone marrow and in the spleen have operated as expected to prevent the expression of DNA binding IgM antibodies, limiting at least one potential adverse self-reactive outcome that could result from the increase in CDR-H3 arginine content caused by the change in D_H_ sequence.

Limitations in the diversity of the CDR-H3 repertoire could also impact the production of autoantibodies and therefore a consideration of the relative repertoires of the mice with different IgH genotypes may be informative. Homozygous wild-type BALB/c mice contain two IgH alleles each encoding 13 D_H_. These gene segments express similar stereotypical patterns of amino acid usage by reading frame with enrichment for tyrosine and a paucity of arginine (**Fig. 1**). Homozygous ΔD-DFL mice contain two IgH alleles, each encoding only one normal D_H_. They express a polyclonal repertoire that mirrors the portion of the WT repertoire using DFL16.1, which contains ~50% fewer arginines in CDR-H3 than the repertoire created by the normal complement of 13 WT D_H_ (**Fig. 2**). These homozygous ΔD-DFL mice, which also serve as a control for limiting the number of D_H_ gene segments to one per IgH allele, consistently demonstrated a lower baseline of both ssDNA and dsDNA-binding IgM and IgG antibodies than mice that contained either WT or ΔD-iD IgH alleles (**Fig. 3**). Heterozygous ΔD-DFL mice contain one IgH allele with 13 WT D_H_ and one IgH allele with a single WT D_H_. They exhibit the same range of sequence diversity as the wild type mice, although they have a proportionately greater representation, or greater heterogeneity, of sequences that were created using DFL16.1. Unlike the homozygous ΔD-DFL mice, several of the heterozygous ΔD-DFL mice exhibited ssDNA-binding IgM production that was higher than baseline, suggesting that antibodies might be using a non-DFL16.1 WT D_H_ (**Fig. 3**).

Homozygous ΔD-iD mice contain two IgH alleles, each encoding one ΔD-iD IgH allele. They express a polyclonal repertoire that is enriched for antigen binding sites encoding arginine in positions that can facilitate dsDNA binding activity (**Fig. 2**) [24,33]. B cells in heterozygous ΔD-iD mice can use both a 13 D_H_ WT IgH allele that creates a wild type tyrosine-enriched repertoire and an IgH allele with one iD D_H_ gene segment that produces a arginine-enriched repertoire, which is normally greatly underrepresented (**Fig. 2**). These mice possess the greatest diversity of CDR-H3 sequence of any of the five studied strains; therefore, the production of DNA-binding IgG in the heterozygous ΔD-iD mice cannot be ascribed to limitations in the potential diversity of the repertoire. It reflects the increased use of arginine-enriched iRF1 sequence contributed by the iD altered D_H_.

In contrast to the apparent suppression of DNA-binding IgM production in the ΔD-iD mice, 40% of the homozygous and heterozygous ΔD-iD mice had elevated serum levels of IgG binding to either ssDNA or dsDNA (**Figs. 3, 4, 6 & 7**). Although the prevalence of DNA binding IgG was greater in heterozygous versus homozygous mice, these differences did not achieve statistical significance. The effect of the ΔD-iD allele was clearly dominant, although penetrance was less than 100% at 12 months of age. It is possible that further aging would have led to increased penetrance, because the pattern of the break in tolerance suggests that age was a factor in DNA binding Ig production. The break in tolerance with time might reflect age-related changes in the regulation or composition of the antibody repertoire, *e*.*g*. a reduction in the ability of somatic selection to curate the repertoire, or an accumulation of mutations in the repertoire that destroys the suppressive effects of light chain editing [45].

### Production of dsDNA binding antibodies is likely T cell driven and could not be attributed to an increase in apoptotic debris or an increase in BLyS levels due to lymphopenia

Trivial explanations for the increase in DNA-binding antibody levels in the D-altered mice include an increase in apoptotic debris that could elicit anti-nuclear antibody production, or a lymphopenia-induced increase in BLyS levels that might permit autoreactive B cells to survive. We found no support for either of these possibilities (**Figs. 8 and 10**).

Production of DNA-specific IgG2a and IgG2b (**Fig. 7**), but not IgM (**Fig. 6**), suggests that acquisition of DNA specificity depends on T cell help, likely as part of a germinal center reaction, although an extra-germinal reaction remains a possibility. This is consistent with observations that dsDNA binding IgG from both lupus-prone mice and humans with lupus is often somatically mutated, suggesting a defect in maintenance of peripheral, rather than central, tolerance [46]. Increasing the prevalence of antibodies with antigen binding sites that can easily acquire dsDNA-binding characteristics due to pre-positioning of arginine residues in CDR-H3 appears to have facilitated production of dsDNA-binding IgG in BALB/c mice, in which mechanisms of peripheral tolerance normally create a barrier to the production of these self-reactive antibodies [47]. This is compatible with our previous finding [48,49] that the prevalence of highly charged, arginine-enriched CDR-H3s in mature, recirculating bone marrow B cells (Hardy fraction F) is higher in MRL and C57BL/6 mice, both of which are more prone to DNA binding antibody production.

### The germline sequence of the D_H_, which is subject to natural selection, can influence the likelihood of autoantibody production

The paucity of glomerular changes in the mice that expressed dsDNA-binding IgG (**Fig. 5**) supports the consensus view that the glomerulonephritis that results in lupus from autoantibody production is a multi-factorial process [50]. However, our mice may offer a means to better understand the role of CDR-H3 content control in the manifestation of lupus. In this light, our results would appear to challenge the classic concept of *Horror autotoxicus*, which holds that the primary mechanisms of selection to prevent the generation of pathogenic self-reactive antibodies are somatic [51]. We would suggest that the first line of defense against the production of potentially pathogenic antibodies is natural selection of germline D_H_ sequence content, which influences the substrate available for somatic mutation and selection post antigen exposure.

## Conclusions

We have previously found evidence for natural selection acting to provide anticipatory protection against a common pathogen [20]. The present work would suggest that natural selection might also provide anticipatory protection against the production of potentially pathogenic, self-reactive antibodies. The binding of a cell surface receptor to its ligand typically transduces a signal that affects the function or fate of the cell. To maintain the specificity of these downstream effects, each receptor typically coevolves with its ligand. At first glance, the B cell receptor would seem to be an exception because its ligand (antigen) binding site appears to vary randomly in sequence and structure on a large scale [2]. However, our work has shown that even CDR-H3, which among the components that comprise the Ig antigen binding site would appear the least tethered to germline determinism by virtue of N addition [52], demonstrates evidence of stereotypy based on specific physico-chemical properties that include enrichment for neutral amino acids such as tyrosine and depletion of highly charged amino acids. These studies suggest that violation of these biases can yield self-reactive humoral responses (this work) as well as immune deficient ones [18,35]. Thus, in addition to antigen-by-antigen discrimination [3,5], there may be a general order to the properties needed by an effective antibody repertoire that depends on natural selection of the range and polyclonal distribution of amino acid content that is permitted or preferred at the center of the antigen binding site.

## Supporting Information

S1 Table Predicted amino acid sequences of CDR-H3 cloned from immature (Fraction E) bone marrow B cells from homozygous TdT-deficient ΔD-iD mice. (DOC)

S2 Table Predicted amino acid sequences of CDR-H3 cloned from bone marrow immature (Fraction E) B cells from homozygous TdT-deficient ΔD-DFL mice. (DOC)
