# Supplementary material for: Violation of an Evolutionarily Conserved Immunoglobulin Diversity Gene Sequence Preference Promotes Production of dsDNA-Specific IgG Antibodies
Source: PLoS One. 2015 Feb 23;10(2):e0118171. doi: 10.1371/journal.pone.0118171 (PMC4338297; doi:10.1371/journal.pone.0118171)
Supplement: S1 Table — (DOC) [file pone.0118171.s001.doc]

**Table S1. Predicted amino acid sequences of CDR-H3 cloned from immature (Fraction E) bone marrow B cells from homozygous TdT deficient *ΔD-iD* mice**

| **ID** | **Base** | **Loop** | **Base** | **#Arg** | **Hydrophobicity** |
| --- | --- | --- | --- | --- | --- |
| 2E-AF303-01 | AR | DYRNHSRS | YAY | 2 | -0.748 |
| 2E-AF303-02 | AS | RNHSRSWY | FDV | 2 | -0.640 |
| 2E-AF303-03 | AR | HRNHSRSY | FDY | 2 | -0.736 |
| 2E-AF303-04 | AR | DRNHSRSYA | MDY | 2 | -0.579 |
| 2E-AF303-05 | AR | HYRNHSRRYY | FDY | 3 | -0.763 |
| 2E-AF303-06 | AR | HRNYYA | MDY | 1 | -0.497 |
| 2E-AF303-07 | AR | YRNHSRSW | FAY | 2 | -0.640 |
| 2E-AF303-08 | AR | HRNHSRS | FDY | 2 | -0.803 |
| 2E-AF303-09 | AR | DRNHSRSWY | FDV | 2 | -0.680 |
| 2E-AF303-10 | AR | HRNHSRSWY | FDV | 2 | -0.670 |
| 2E-AF303-11 | AR | DHRNHSRSY | FDV | 2 | -0.766 |
| 2E-AF303-12 | AR | HRNYYA | MDY | 1 | -0.497 |

ID, sequence identifier. Base, the predicted amino acid sequence of the CDR-H3 base. Loop, the predicted amino acid sequence of the CDR-H3 loop. #Arg, number of arginines in the CDR-H3 loop. Hydrophobicity, the average, normalized Kyte-Doolittle hydrophobicity of the CDR-H3 loop.
