# Supplementary material for: Violation of an Evolutionarily Conserved Immunoglobulin Diversity Gene Sequence Preference Promotes Production of dsDNA-Specific IgG Antibodies
Source: PLoS One. 2015 Feb 23;10(2):e0118171. doi: 10.1371/journal.pone.0118171 (PMC4338297; doi:10.1371/journal.pone.0118171)
Supplement: S2 Table — (DOC) [file pone.0118171.s002.doc]

**Table S2. Predicted amino acid sequences of CDR-H3 cloned from bone marrow immature (Fraction E) B cells from homozygous TdT deficient *ΔD-DFL* mice**

| **ID** | **Base** | **Loop** | **Base** | **#Arg** | **Hydrophobicity** |
| --- | --- | --- | --- | --- | --- |
| 9126 | AR | HYYGSSY | FDY | 0 | -0.270 |
| 9127 | AR | HYYY | FDY | 0 | -0.430 |
| 9128 | AS | YYGSSYWY | FDV | 0 | -0.174 |
| 9129 | AR | HYGSSY | FDY | 0 | -0.270 |
| 9130 | AR | HYYGSSWY | FDV | 0 | -0.254 |
| 9131 | AR | DYGSSY | FDY | 0 | -0.285 |
| 9132 | AS | YYGSSYWY | FDV | 0 | -0.174 |
| 9133 | AR | HYYGSSSYWY | FDV | 0 | -0.240 |
| 9134 | AR | HDYGSYWY | FDV | 0 | -0.366 |
| 9135 | AR | HYYGSSWY | FDV | 0 | -0.254 |
| 9136 | AR | LYYGSSYY | FDY | 0 | 0.019 |
| 9137 | AR | HYYGSSYA | MDY | 0 | -0.140 |
| 9138 | AR | HYY | GDY | 0 | -0.483 |
| 9139 | AR | FITTVVGY | FDV | 0 | 0.703 |
| 9140 | AS | YYGSSW | FAY | 0 | -0.142 |
| 9141 | AR | DYYGSSYWY | FDV | 0 | -0.266 |
| 9142 | AR | HYYGSSYWY | FDV | 0 | -0.256 |
| 9143 | AS | YYGSS | FDY | 0 | -0.142 |
| 9144 | AR | HYYGSSY | VDY | 0 | -0.270 |
| 9145 | AS | YYGSSYWY | FDV | 0 | -0.174 |
| 9146 | AT | TVVDWY | FDV | 0 | 0.287 |
| 9147 | AR | HYYGSSYWY | FDV | 0 | -0.256 |
| 9149 | AR | DYYGSSWY | FDV | 0 | -0.265 |
| 9150 | AR | HYYGS | FAY | 0 | -0.304 |
| 9151 | AR | DYYGSSYWY | FDV | 0 | -0.266 |
| 9152 | AR | HGSSYY | FDY | 0 | -0.270 |
| 9153 | AR | HYYGSSYY | FDY | 0 | -0.270 |
| 9154 | AR | YGSSYYYA | MDY | 0 | -0.060 |
| 9155 | AT | TVVDWY | FDV | 0 | 0.287 |
| 9156 | AR | HYYGSSYA | MDY | 0 | -0.140 |
| 9157 | AR | DYYYGSSYD | FDV | 0 | -0.361 |
| 9158 | AR | HGSSYYWY | FDV | 0 | -0.254 |
| 9159 | AR | HYYGSSYY | FDY | 0 | -0.270 |
| 9160 | AR | DYYGSSYWY | FDV | 0 | -0.266 |
| 9161 | AR | HYGSSYWY | FDV | 0 | -0.254 |
| 9162 | AR | HYYGSSWY | FDV | 0 | -0.254 |
| 9163 | AR | LLRWY | FDV | 1 | 0.218 |
| 9164 | AR | HYYGSSWY | FDV | 0 | -0.254 |
| 9165 | AR | HYGSSYWY | FDV | 0 | -0.254 |
| 9166 | AR | HYGSRYY | FDY | 1 | -0.441 |
| 9167 | AR | LYYGSSYY | FDY | 0 | 0.019 |
| 9168 | AR | YYYG | FDY | 0 | -0.195 |
| 9169 | AR | DSYYA | MDY | 0 | -0.174 |
| 9170 | AR | HYYGSSYWY | FDV | 0 | -0.256 |
| 9171 | AR | HYYGSSY | VDY | 0 | -0.270 |
| 9172 | AR | HYYGSNY | FDY | 0 | -0.399 |

ID, sequence identifier. Base, the predicted amino acid sequence of the CDR-H3 base. Loop, the predicted amino acid sequence of the CDR-H3 loop. #Arg, number of arginines in the CDR-H3 loop. Hydrophobicity, the average, normalized Kyte-Doolittle hydrophobicity of the CDR-H3 loop.
